# Hospital physicians’ experiences and reflections on their work and role in relation to older patients’ pathways - a qualitative study in two Norwegian hospitals

**DOI:** 10.1186/s12913-022-07846-1

**Published:** 2022-04-05

**Authors:** Ann-Chatrin Linqvist Leonardsen, Anne Werner, Hilde Lurås, Anne-Kari Johannessen

**Affiliations:** 1 Østfold University College, (PB) 700, 1757 Halden, Norway; 2 Østfold Hospital Trust, (PB) 300, 1712 Grålum, Norway; 3 Health Services Research Unit, Akershus University Hospital, 1000, 1478 Lørenskog, Norway; 4 Institute of Clinical Medicine, Campus Ahus, University of Oslo, Oslo, Norway; 5 Department of Nursing and Health Promotion, OsloMet – Oslo Metropolitan University, P.O. Box 4, St. Olavs plass, Oslo, Norway

**Keywords:** Hospital physicians, Older patients, Hospital admission, Hospital discharge, Interview

## Abstract

**Background:**

Older patients are the most frequent users of initial hospital admissions and readmissions. Both hospital admission and discharge require communication and coordination between healthcare professionals within the hospital, and between professionals in hospitals and primary healthcare. We have identified few studies exploring hospital physicians’ perspectives on older patients’ pathways in the interface between hospital and primary healthcare services. The aim of this study was to explore hospital physicians’ experiences and reflections on their work and role in relation to older patients’ pathways between hospital and primary healthcare. Specifically, we focused on the challenges they faced and how they dealt with these in relation to admission and discharge, and their suggestions for service improvements that could facilitate older patients’ pathway.

**Methods:**

We used a qualitative approach, conducting individual in-depth interviews with 18 hospital physicians from two hospitals in eastern Norway. Data were analyzed using systematic text condensation, in line with a four-step prosedure developed by Malterud.

**Results:**

The participants emphasized challenges in the communication about patients across the two service levels. Moreover, they described being in a squeeze between prioritizing patients and trying to ensure a proper flow of patients through the hospital wards, but with restricted possibilities to influence on the admissions. They also described a frustration regarding the lack of influence on the healthcare delivery after discharge. The participants had various suggestions for service improvements which might be beneficial to older patients.

**Conclusions:**

The results demonstrate that the hospital physicians perceived being squeezed between professional autonomy and limited capacity at the hospital, and between their medical judgement as a specialist and their power to decide on hospital admissions for old patients and also on the delivery of health care services to patients after discharge.

**Supplementary Information:**

The online version contains supplementary material available at 10.1186/s12913-022-07846-1.

## Background

The proportion of older persons in Europe is increasing. By 2070, 30.3% of the population is expected to be 65 years or older and 13.2% is expected to be 80 years or older [1]. Simultaneously there has been a decrease in the number of hospital beds, and hospital physicians are expected to discharge patients early to improve hospital efficiency [2, 3]. As a consequence, there is an increased need to offer accessible health- and care services prior to and after hospitalization, as well as a need to implement alternatives to hospitalization [4–6].

Older patients, defined as persons 60 years or above [7], are the most frequent users both of initial hospital admissions and readmissions [8]. Hospital discharge has been described as a complex system involving dynamic and multidirectional patterns of knowledge sharing between multiple stakeholders [9]. Studies indicate that patients are discharged with more complex medical conditions than they were previously, and discharge are sometimes perceived as premature [10, 11]. For older patients characterized by multimorbidity, functional decline and complex medical regimens the transition between hospitals and primary healthcare is associated with medication discrepancies [12, 13], insufficient medical information [14, 15], and uncoordinated care [16]. It is also reported a high hospital readmission rate among old patients [3, 16, 17]. Both hospital admission and discharge require communication and coordination between healthcare professionals within the hospital, and between professionals in hospitals and primary healthcare [18]. Such collaboration has been shown to be challenging, due to differences in involved personnel (e.g. nurses, physicians), a division between specialist and generalist services (e.g. two governmental levels), as well as an uncertainty related to who is responsible for the patient [19–21].

In Norway the healthcare system is organized within two different governmental levels. Stateowned hospitals provide specialized medical services, while the municipalities are responsible for organising primary healthcare, short- and long-term care and home nursing. Every inhabitant is listed with a primary care physician who hold the medical responsibility for patients on his or her list. Hospitals and primary healthcare are subject to different funding systems, laws and central regulations, and also to different electronic patient journal systems. The Norwegian Coordination reform [22], which was implemented from 2012, focused on improving coordination and collaboration between healthcare levels. Some of the initatives were to establish municipal acute wards as alternatives to hospitalization, introducing penalty fees for municipalities not ready to receive patients discharged from hospital, as well as statutory collaboration agreements between hospitals and municipalities. A majority of the Norwegian municipalities are set up with a provider split model implying a distinction between those who assess the need for services after hospital discharge (purchaser office) and those who provide the services [23]. To ensure transfer of sufficient and relevant information between hospitals and primary healthcare, electronic dialogue messages have been introduced as a tool to improve collaboration. The dialogue messages comprise a set of standardised messages to support the admission, assessment/treatment, and discharge phases of a hospital stay [24].

Earlier studies have explored primary healthcare professionals’ perspectives on older patients’ pathways within the healthcare system [21, 25, 26]. A qualitative study that included primary care nurses’ and hospital physicians’ perspectives on the transition from hospital to home concluded that effective communication between professionals across different healthcare institutions and different governmental levels is essential to reduce the readmission rate and improve safety and continuity of care [27]. Beyond these, we have identified few studies exploring hospital physicians’ perspectives on older patients’ pathways in the interface between hospital and primary healthcare services.

The aim of this study was to explore hospital physicians’ experiences and reflections on their work and role in relation to older patients’ pathways between hospital and primary healthcare. Specifically, we aimed at exploring: 1) challenges they faced and how they dealt with these in relation to admission and discharge, and 2) their suggestions for possible initiatives that could improve the service delivery and the patient pathway for older patients.

## Material and methods

Qualitative methods are especially appropriate for answering questions of *why* something is observed, to study of the nature of a phenomena, as well as to take research participants’ own experiences, views and meaning making as the starting point [28, 29]. Hence, to explore hospital physicians’ experiences and reflections on their work and role in older patients’ pathways we used a qualitative approach, conducting individual in-depth interviews with hospital physicians. The study adheres to the Consolidated Criteria for Reporting Qualitative Research (COREQ) guidelines [30].

### Setting and participants

The study was conducted in two hospital catchment areas in the eastern part of Norway. Hospital 1 is a university hospital with 1000 beds, and a catchment area of 600.000 inhabitants. Hospital 2 is a local hospital with 700 beds, and a cathcment area of 320.000 inhabitants.

We used a purposive sampling strategy covering hospital physicians who work or had previously worked with older patients in a hospital ward. We contacted clinical leaders who recommended participants to the study. Potential participants were contacted via email, including information about the study and the consent to participate form.

### Reflexivity

The research team included two nurse anesthetists (NA), a medical sociologist and a healtheconomist with background as a physiotherapist (all female), with a ph.d. and currently working as academics and researchers. Three were experienced in qualitative research methodology, and all four experienced in the field of transitions between health service levels. Different researchers might access different, although equally valid, representations of the situations studied. Hence, a method of reflexivity was included to actively include the researchers’ impressions and preconsumptions [31]. As such, the researchers wrote down reflexivity notes, including thoughts and preconsumptions before and after each interview, as well as impressions about the context, nonverbal expressions and interaction between the researcher and the participant from the conversations. These notes were included throughout the analysis process. For instance, three of the researchers (NAs) had several years of experience from working in hospitals, and had a preconsumption that hospital physicians’ main concern was medical treatment. Through analysis and discussions in the research group throughout the current study it became obvious that hospital physicians were conserned about the whole patient pathway.

### Data collection

An interview guide (appendix 1) was developed based on earlier studies on admission to and discharge from hospital for older patients, as well as through discussions between the authors. The semi-structured interviews were open-ended, and dealt with hospital physicians’ experiences with their work related to hospital admission and discharge, and their reflections about initiatives that could improve the service delivery and the patient pathway for older patients.

Individual interviews were conducted in the period August to December 2020 by one of the researchers (ACL, AW or AKJ), in a meeting room in each of the respective hospitals. The interviews lasted from approximately 30 min to 1 h, on average 40 min. All interviews were digitally recorded and transcribed verbatim by the researcher who conducted the interview.

### Analysis

Transcripts of interviews constituted our data, which was analyzed using systematic text condensation, a descriptive and explorative, qualitative procedure for thematic analysis across individual participants, as developed by Malterud [32]. The method represents a pragmatic approach, inspired by phenomenological ideas, searching for the participants’ subjective experiences. This also includes a process of intersubjectivity, reflexivity, and feasibility, while maintaining a responsible level of methodological rigour. The procedure consists of the following steps: 1) total impression - from chaos to themes; 2) identifying and sorting meaning units - from themes to codes; 3) condensation - from code to meaning; 4) synthesizing - from condensation to descriptions and concepts. The analysis followed these steps, as a process constantly moving back and forth between the steps.

The first step included all authors reading the transcripts to get an overall impression of hospital physicians’ experiences, recognizing nine preliminary themes. These preliminary themes were guided by the questions in the interview guide. Moreover, reflexivity notes were included in the discussions, making all authors aware of preconsumptions possibly impacting the analysis. In step two the nine prelimiary themes were collapsed based on discussion between all authors, resulting in three main code groups; challenges in relation to 1) ‘admission’, 2) ‘discharge’ and 3) ‘the hospital physicians’ suggestions for service improvements’. Here, we identified meaning units, representing different aspects of the individual physicians’ experiences from their clinical work, including their collaboration with primary healthcare. In step three we abstracted the content of each meaning unit within the three code groups. Here, we established subgroups within each code group, condensing the content of each group and identifying illustrative quotations from the interviews. In step four, we synthesized the condensates from each code group and subgroup, and presented a reconceptualized description of each category relating to the aim of the study. Here, the final text in the result section was also validated against the interviews. Table 1 gives an example of the analysis process.

**Table 1:** Example of the analysis process

Meaning unit	Code group	Subgroup	Category	Illustrative quotes
«We want to assume what is the appropriate further path before discharge … But, it is affected by the capacity (in hospital) … It may be a day sooner if we have four patients in the corridor … If you think it is safe, then that’s the way it is» (P 3)	A calculated risk	Proper pathways for older patients	Suggestions for future organization of healthcare services to older patients	“It has to bee an improvement … At least an assumption of what is wrong. And if we don’t know, at least that the patients’ condition is improving” (P 1) “It has to be medically sound to discharge them. And that depends on where we discharge them to. If they cannot stay at home, but need to stay in a nursing home or an institution … It depends on whether we can state that we cannot offer better services in an acute wards, and therefore discharge them to a nursing home” (P 16)

During the analysis, the authors routinely met to discuss preliminary themes developed from the meaning text unites of the transcribed interviews and the coding of the data, including the reflexivity notes.

### Ethical approval and consent to participate

The study was based on research ethical guidelines as presented in the Declaration of Helsinki, and on anonymity, written, informed consent and the right to withdraw without any negative consequences for the participant [33]. The privacy legislation authority at Akershus University Hospital approved the study (ref. 2017_058). The Regional Committee for Medical & Health Research Ethics, Section A, South East Norway, found the Research Project to be outside the remit of the Act on Medical and Health Research (ref.: 2016/2277a, IRB00001871).

## Results

The sample of hospital physicians included a total of 18 participants; nine women and nine men between 29 and 69 years old (average age 42.1 years). Their experiences from hospital work varied from 1 year to more than 30 years. Table 2 gives a description of the study participants.

**Table 2:** Descriptives of the study participants (*N* = 18)

**Age (in years)**	***n*=**
< 30	2
30–39	4
40–49	7
50–59	3
60–69	2
**Gender**	***n*=**
Male	9
Female	9
**Speciality**	***n*=**
Infection medicine	1
Neurology	3
Surgery	3
Geriatrics	6
Internal medicine	5
**Length of experience in hospital (in years)**	***n*=**
0–5	2
6–15	7
16–25	4
26–30	2
30 +	3

We identified three categories including subgroups: 1) Searching for efficient information sharing, 2) Handling complexity in patient needs, with subgroups a) trying to avoid admission of «unsuitable patients», and b) facilitating proper flow of patients, and 3) Suggestions for future organization of healthcare services to older patients, with subgroups a) proper pathways for older patients, and b) suggestions for service improvements.

### Searching for efficient information sharing

The hospital physicians reported various experiences regarding communicating with primary care physicians on admission and discharge of older patients. Even though they initially reported the communication to be good, they emphasized challenges. Insufficient information about hospitalized patients was regarded «a huge challenge». Regularly the hospital ward had to call the primary care physicians’ office to gather information about hospitalized patients’ list of prescriptions, and to contact family caregivers or home nursing to get updated information about the patient’s functional status prior to hospitalization. Participant 6 emphasized the work invested in obtaining necessary information:

«Sometimes, we know very little about the patients’ level of functioning. This is important, because they are often confused, weak, seem malnutritioned, and we don’t know if this has been ongoing for a while or not.»

The participants also reported that they had limited communication with primary care physicians during hospital admission and discharge. Participant 18 said: «It’s no close contact with the primary care physicians - no communication between us actually.»

The hospital physicians thought limited communication could impede clarifications of important medical issues regarding the patients. To get in touch with the primary care physician by phone was perceived a time-consuming procedure which often failed, as one Participant 12 stated: «I can’t remember the last time I called a primary care physician. It’s like they are doing their [work], and we are doing ours.»

Some also stressed that they did not have capacity to answer phone calls from primary care physicians, since «the day is already filled up with tasks».

Mostly, the communication between hospital and primary healthcare happened through the newly introduced electronic platform, the dialogue messages. The majority of the participants pointed to various challenges regarding the dialogue messages. Participant 1 said they spent much time and energy on writing these messages at the cost of patient treatment:

«I would say about 90% [of the communication] is through the digital messages. Sometimes we use the telephone, but then its’ most often nurses calling nurses (…). Digital messages take so much time, and there’s also a risk of misinterpretations of written text. We have examples of both ten and twenty messages back and forth during one day, and this occupies our nurses and also the nurses in primary healthcare.»

The few telephone calls the hospital physicians received from primary care physicians were often about getting «a second opinion» on their assessment of the patient before admission or after discharge. This was either in relation to medication, treatment or regarding difficulties to diagnose the patient due to limited diagnostic opportunities in primary health care.

### Handling complexity in patient needs

Most of the participants expressed a worry about admitting patients that rather could have been treated in primary healthcare. This was related to what could be a misjudgment of the patients’ condition, and to the fact that they felt responsible for utilizing the hospital capacity too much.

#### Trying to avoid admission of «unsuitable patients»

Some of the participants expressed that primary care physicians often interpret the acute frailty of older patients as more dramatic than the hospital physicians do. Yet, the participants were aware of the difficult medical responsibilities the primary care physicians have. Participant 5 stated: «I think we should rather assess too many patients to the hospital than too few.»

Nevertheless, all the participants stressed the importance of avoiding admission if not «absolutely necessary». Participant 4 described how he dealt with this:

«It’s about asking questions, trying to map out what is the reason why they want to admit the patient or want an assessment, and it is about considering whether this has to be done today, trying to help them to make a decision.»

The participants had a clear understanding of which patients that should not be hospitalized. Examples were patients in a terminal phase of a disease, patients with chronic conditions, or patients diagnosed with cognitive deficiency living permanent in a nursing home. However, the hospital physicians also pointed to the limited power they have to influence the decisions, since primary care physicians have a legal right to admit patients to hospitals.

Many of the participants suggested that the primary care physicians more frequent should have called them to discuss whether hospitalization was necessary or not. Participant 1 described a particular situation: «A patient with Alzheimers’ came in. He was agitated and could not sleep, and they had given him four different drugs to calm him down. After inspection we inserted a urinary catheter, and one litre urine came. Then he slept all night, and left the morning after.»

It was obvious to the hospital physician that this should have been treated in primary healthcare, but also that the hospital physicians could have an advisory role in such situations.

#### Facilitating proper flow of patients

The participants described the hospital as «overloaded with patients» and that they had to ensure a proper flow of patients through the wards. Some of the participants also described a continuous «pressure» on discharging patients. Even though participant 2 felt it «*unhuman to put such a pressure on the professionals*», she said: *«*I use to say that we don’t merely have responsibility for the 22, 23 or 18 [patients] in our ward. We also have a responsibility for those not coming to us. We are responsible for the whole county.»

Many of the participants pointed to dilemmas of prioritizing between patients, i.e. whom and when to discharge. They described discharging patients as «an everyday risk» due to the many potential medical pitfalls. Sometimes patients had to be readmitted shortly after discharge even though the discharge had been thoroughly considered and discussed in the medical team at the hospital. Participant 12 argued that early discharge is «a calculated risk».

The hospital physicians emphasized that the patients’ condition needed to be improved before discharge, and particularly that the patients must be able to «stand on their feet and walk to the toilet», as two of the participants argued. Some of the participants even stated that they occasionally «held patient back one or two nights» although the patient was assessed ready for discharge.

### Suggestions for future organization of healthcare services to older patients

All of the participants had a genuine interest of achieving healthcare services that were to the best of the older patients. However, they worried about whether the services offered to date were appropriate. Participants obviously had reflected about how services could be improved, and gave several examples of this.

#### Proper pathways for older patients

The participants were worried about the national health authorities’ policy of strengthening municipal healthcare at the expense of institutional beds in hospital, and the municipalities’ policy of strengthening home-based care at the expense of reducing nursing home beds. Participant 8 reflected on the development in the healthcare services in this way:

«Patients are getting older and sicker with more comorbidities. They need longer stays, meanwhile we try to cut the length of stay. They need more medical treatment in addition to care, and they often have complex conditions and co-existing complex diseases, which demands different medical specialties. Often they do not recover.»

Several of the participants described older patients not suffering from strict somatic diseases. They referred to cognitive impairment, psychological and/or social problems, loss of partner or lack of social network, poor living conditions or nutrition related problems. Participant 4 illustrated the challenges as follow: «One category is when it does not work out at home, and they have managed until a certain point, and when it is not possible to compensate anymore. They may not be very sick right here and now, but they are too sick to stay at home.»

The participants stated that they did not know what the patients were offered from primary healthcare after discharge, and some of them were concerned about the variation in service delivery between municipalities. Some also stressed that they did not have a complete overview of the available healthcare services in the residents’ municipalities. For instance, a participant mentioned an episode where she assumed that there were physicians present on a daily basis in the healthcare institution she had referred the patient to, but was «a bit shocked» when she recognized that this was not the case.

Situations where personel from the purchaser office in the municipality visited the hospitalized patient to assess the need for further services after discharge, were described as frustrating. Participant 6 said: «When it is absolutely obvious that the patient will not be able to manage herself at home, and the municipality [the purchaser] come on assessement visit [to the patient] rather than communicating directely with us – that surprises me. I do think it is kind of a weird re-examinination of our competence*.»*

Some of the participants pointed to the purchaser office as the one they had most quarrels and conflicts with when discharging patients. In cases when family caregivers had denied taking the patient home, the hospital physicians felt as being caught in a disagreement between the caregiver and the purchaser office in the municipality.

#### Suggestions for service improvements

Many of the participants had suggestions regarding how to improve the service delivery that particularly would be beneficial to older patients. The key message was a necessity to strengthen and improve the healthcare services as a whole. The participants recommended more coordinated and seamless collaboration between hospital and primary healthcare to the most fragile old patients. Participant 3 said: «Some could probably have avoided a short-term stay in a nursing home if the hospital could have kept them one, two, [or] 3 days longer, and that would spare capasity outside [in the municipalities].»

Many of the participants also suggested several improvements both within the hospital and in the interface between the hospital and primary healthcare. The participants argued that there is an urgent need of establishing more intermediate care beds and rehabilitation units, both outpatient and 24-h beds. They emphasized a need to increase the acute geriatric and the psychiatric competence in the service delivery. Examples mentioned were to include geriatricians in the emergency ward, and geriatric teams that could support the hospital wards. Establishing ambulant geriatric teams following up on older persons discharged from hospital, or geriatric positions circulating between hospitals and municipalities were also suggested. More dedicated time to inform patients and family caregivers about treatment plans and follow-up of patients was also highlighted. Participant 5 said: «I always wish for more time with the patient. I feel most of my work is in front of a computer, completing referrals. [I want] to sit down [with the patient] and go through what has happened during the hospital stay.»

Direct communication between hospital physicians and primary care physicians was also assumed to be an important improvement. Clarification of medical matters, such as deciding who is to be responsible for updating the prescription list, was seen to be easier through a direct dialogue with the primary care physician than using electronic dialogue messages. Another important task to solve together with primary healthcare was to develop and implement standardized medical treatment plans to old patients with complex medical conditions.

## Discussion

The results demonstrate the discrepancy the hospital physicians experienced regarding the hospital’s capacity to admit patients and old patients’ overall healthcare needs. The participants emphasized challenges in the communication about patients across the two service levels. Moreover, they described being in a squeeze between prioritizing patients and trying to ensure a proper flow of patients through the hospital wards, but with restricted possibilities to influence on the admissions. They also described a frustration regarding the lack of influence on the healthcare delivery after discharge. The participants had, however, various suggestions for service improvements which might be beneficial to older patients.

### Challenging (electronic) communication

The results indicate that direct communication between hospital physicians and primary care physicians about patients was scarce. Mostly, the communication happened through electronic written information, which was perceived as helpful, but also as time-consuming. Moreover, this increased the risk of misunderstandings and led to a consecutive need for further dialogues to clarify the patients’ functional status, goals for the medical treatment, expectations about e.g. recovery. Even though studies among physicians working in hospitals or primary care show that electronic messages are time-saving [24, 34], studies also have shown that telephone calls and face-to-face communication are preferred because of the immediate need to sort out inquiries on patients [35, 36].

Furthermoreover, the participants in our study requested more time for personal communication with primary care physicians. Yet, they also emphasized that there were neither time for face-to-face collaboration nor were primary care physicians easy to reach on telephone. These findings confirm previous research that have demonstrated that healthcare professionals from hospital and municipalities invest much time and work to obtain an overview of a patient’s situation [37]. However, one study indicated that there is no relationship between several aspects of communication between hospital and primary care physicians and associated adverse clinical outcomes for patients [38]. Another study found that an encrypted e-mail could be used as a tool to obtain useful additional medical and psychosocial information between hospital and primary care physicians [39]. Hence, efficient ways to exchange information between hospitals and primary care still needs to be explored.

The participants in our study also revealed a tension, and often a disagreement, between hospital professionals’ and the municipal purchasers’ views regarding patients’ need for service delivery after discharge. The described tension may indicate that physicians experienced a lack of autonomy in their decisions regarding discharge. This is in line with studies on primary healthcare professional’s experiences from collaboration with the purchasers’ office [40, 41]. Salvatore et al. [42] suggest that organizations should support the organizational and economic autonomy of their physicians to support development of an organizational identity, which has been considered a source of positive employee outcomes [43].

There is an extensive literature on collaboration in healthcare, particularly related to older patients’ pathways, transitional care and discharge processes [8, 24, 44]. Studies have shown that interprofessional communication about patients with several comorbidities and polypharmacy issues is challenging and complex [37, 45]. Partnership and the sharing of goals and responsibilities have been pointed to as important factors explaining good collaboration [46]. Correspondingly, factors explaining difficulties in the collaboration are different goals, tasks, responsibilities and clinical roles [19, 47]. Hence, providers with conflicting responsibilities seem to have problems viewing situations from the other providers’ perspectives. Consequently, focusing on improving collaboration between hospital physicians and primary care physicians seems to be beneficial to ensure quality in older patients’ pathways between hospital and primary healthcare.

### Patient flow in hospital versus continuous needs among municipal residents

Our results show that the hospital physicians worried about older patients’ pathways due to the complexity of their health conditions and their need for comprehensive healthcare services. Even though they gave medical advice to primary care physicians they reported limited possibility to influence on the inflow of patients to the hospital and the service delivery offered after discharge. In addition, they reported to have limited knowledge about the available healthcare resources in the municipalities generally and the healthcare services specificially.

Our study support research showing that patients are discharged earlier with more complex medical conditions than previously, and that discharge sometimes is perceived as premature [10, 11]. Glette et al. [10] also reported that insufficient capacity in the hospital resulted in pressure to discharge patients, and that the primary healthcare services was not always able to meet the patients’ healthcare needs. Hence, early hospital discharge, poor communication between healthcare services and inadequacies in the discharge process were perceived to affect hospital readmissions.

Our results indicate that the hospital physicians were squeeczed between professional autonomy and limited capacity, and between medical judgement and limited poer or authority to decide which patients who need hospital admission and what the patients should be offered after discharge. This is confirmed by Andri & Kyriakidou [48], who reported that there was a clear pressure towards constraining health professionals’ power to self-define their relationship with patients and towards curtailing their discretionary domain over the use of resources. The physisians’ medical autonomy was weakened due to bureaucratic control of the allocation of resources. A 2017 review [49] found that increased decision space, defined as degree of choice and transfer of decision-making capasity, reduce the bureaucracy surrounding decision-making in the health sector.

### Improvement of services for older people

The hospital physicians recommended more coordinated and seamless collaboration between hospital and primary healthcare to the most fragile old patients. This is in line with the intentions of the Norwegian Coordination Reform [22] that aimed at improving collaboration and coordination of care pathways for patients with complex needs, of which the elderly patients are a core group. Our results indicate that these issues still need emphasis almost 10 years after the implementation of the reform.

Moreover, the hospital physicians recommended establishment of geriatric treatment teams, both in hospital and in primary care. Liu et al. [50] found an association between the introduction of an interprofessional geriatric assessment team, and a lower hospital admission rate, due to avoiding the risk for functional decline, complications and adverse events associated to hospitalization. Several studies show that geriatric competence prevents in-hospital functional decline, decreases mortality and institutionalization rates and results in shorter length of stays, a lower incidence of delirium, fewer falls, more discharges to home and lower costs [51].

The need for integration of healthcare services and collaboration across organizational boundaries is highlighted as a major challenge within healtcare in many countries [47, 52]. In the effort to deliver integrated care, tension as well as a variety of cultures among different healthcare institutions and professionals has been described [53]. The fact that the Norwegian healthcare system is organized in two different governmental levels with different organizational, financial and legal constraints, make the collaboration between hospital and primary healthcare difficult. To contribute to a more seamless collaboration and communication across organizations and disciplines in healthcare Doessing and Burau [53] suggest that such boundaries should be eliminated. Olsen et al. [54] found that healthcare providers experience a conflict between market and public management logics’ ideals of equality, standardization, and efficiency, and health care professionals’ ideals of individualized care. They emphasize the need for situated encounters to enable knowledge sharing between healthcare personnel at different healthcare levels, and also the importance of including older patient’s perspective on the pathway [55].

### Strengths and limitations

Qualitative approaches limit the opportunity to generalize findings. The study draws on interviews with a relatively small sample of 18 hospital physicians recruited from two different hospitals in the eastern part of Norway. We applied a purposive sampling strategy covering participants representing different wards and positions within the hospitals. This gave a broad picture and insights from physicians in different wards and hospitals. In addition, our findings are supported by international research findings, which also increase the transferability of our findings.

The credibility of our findings is supported by the transparency in the presentation of analysis and results. In addition, we aimed at challenging our own preconceptions through writing reflexivity notes before and after each interview, which were kept close throughout the analysis and interpretation of findings. The researchers have solid knowledge from and about patient treatment and mechanisms in transitional care in both hospital and primary care settings, which was a strength when developing the interview guide.

We did not identify any differences in experiences relating to admission, discharge or areas of improvement between participants from different hospital departments. However, adding questions relating to what kind of department the participant worked in may have revealed such differences.

## Conclusion and implications

This study adds knowledge about hospital physicians’ perspectives on older patients’ pathways between hospital and primary healthcare services. Our findings uncover the hospital physicians’ challenging position, being squeezed between professional autonomy and limited capacity in both hospital and primary healthcare, and between their medical judgement of what older patients need and the available services. Hence, it seems that a healthcare system organized on two governmental levels subject to different laws, regulations and funding systems is challenging, and that it creates obstacles to pathways for older patients. Digital platforms and electronic communication seem not to fully compensate for the lack of direct communication between hospitals and primary care, and the need for better alternatives seems obvious.

## Supplementary Information

**Additional file 1.**

## Data Availability

Data material generated and/or analyzed during the current study are not public available from the corresponding authors due to local ownership of data, but aggregated data are available from the corresponding author upon reasonable request.
