# Supplementary material for: Hospital physicians’ experiences and reflections on their work and role in relation to older patients’ pathways - a qualitative study in two Norwegian hospitals
Source: BMC Health Serv Res. 2022 Apr 5;22:443. doi: 10.1186/s12913-022-07846-1 (PMC8981867; doi:10.1186/s12913-022-07846-1)
Supplement: Supplementary file 1 — Additional file 1. [file 12913_2022_7846_MOESM1_ESM.docx]

**Interview guide**

**1. The old patient**

Please describe the patient groups you are primarily in contact with. Describe their challenges and needs

**2. Collaboration in relation to hospital admission**

- When in contact with a PCP regarding a hospital admission, which criteria do you emphasize? Follow up: Which services can the hospital ensure that can not be ensured in the municipalities/by municipal healthcare personnel?
- How do you experience collaboration with tadmitting physicians? Follow up: Can you please describe a situation including a positive collaboration? Or a negative?
- Are there differences based on where patients are admitted from? E.g. from home or from a nursing home

**3. Collaboration in relation to hospital discharge**

- Which criteria do you find essential for patients to be ready for discharge?
- What has to be arranged to ensure a safe discharge of patients?
- Is there something you find challenging in relation to discharge of old patients?
- How do you collaborate with PCPs or other healthcare personnel after discharge? Follow up: what do this collaboration consist of?
- Which assessments do you do do regarding healthcare services to patients after discharge?
- In your opinion: are there differences regarding medical treatment and care if patients are discharged to home-based nursing, nursing homes or intermediate care units?

**4. Alternatives to hospitalization and organization of healthcare services**

- In your opinion: are there patients that do not need hospitalization? Please elaborate.
- What has to be in place for patients to be able to receive medical treatment outside hospital instead of being hospitalized? Follow up: Diagnostic opportunities? Competence?
- Municipal healthcare services- do you have any point-of-view on services and organization? (e.g. capasity, decentralization, municipal services versus hospitalization)

**Finisher**

- If you could decide, how would old patients’ pathways be organized and fascilitated?
